# Attitudes of Turkish Medical and Law Students towards the Organ Donation

**Published:** 2015-02-01

**Authors:** M. Sağiroğlu, O. Günay, E. Balci

**Affiliations:** Erciyes University Medical Faculty Department of Public Health, Kayseri, Turkey

**Keywords:** Faculty, medical, Law faculty, Student, Tissue and organ procurement, Transplantation, Attitudes

## Abstract

Background: Attitudes of medical and law personnel towards organ donation are very important.

Objective: To compare the attitudes of the medical and law students towards organ donation.

Methods: 498 students in the 1^st^ and 4^th^ grades of the medical and law faculties of Erciyes University, Kayseri, Turkey, in 2011–12 academic year, were included in this study. A questionnaire consisting of 31 questions on socio-demographic characteristics of the students and their attitudes towards organ donation and transplantation was administered to the participants.

Results: The percentage of the students who donated organs was 1%. Approximately, 48% of the medical students and 34% of the law students stated that they think to donate organs. The percentage of the students with a positive attitude towards organ donation was found significantly higher among the medical students than the law students, and higher among the 4th grade compared to the 1^st^ grade.

Conclusion: The percentages of the students who have donated organs and think to donate are rather low. Medical students’ attitude towards organ donation was more positive than the law students.

## INTRODUCTION

Organ failure is one of the important worldwide public health problems. Transplantation is an effective therapeutic modality for irreversible organ failure [1]. Organs and tissues that are necessary for transplantation can be achieved from the live donors or from cadaver [2, 3]. Nowadays, transplantation can be performed for many organs and tissues, such as heart, liver, kidney, pancreas, bone marrow, blood, skin and cornea [4].

In Turkey, transplantation is practiced according to a law the so-called “The Law on Organ Donation,” ratified in 1979 and revised in 1982. In this law, it has been stated that organs or tissues can be taken from a cadaver if there is a written testament of the deceased or with the consent of the relatives. Organs and tissues cannot be taken if there is a statement of the deceased, indicating he or she was opposed to organ donation [5]. 

Insufficiency of organ donation is a common problem all over the world. Waiting lists for organs are getting longer every day. In contrast, organ supply remains relatively constant. In Turkey, like other countries, for lack of transplants, many patients in need of transplant wait for a long period and many die every year [6, 7]. The legal and health care staffs should be responsive and knowledgeable about organ donation because of the legal and medical aspects of the problem. In the meantime, the society as a whole should have sufficient knowledge about and positive attitudes towards organ donation. In the medical literature, there are a lot of articles related to the knowledge, attitudes and practices of the health professionals and health-related school students [8–16]. Most of these articles are on the physicians and medical students. However, we could not find any study related to the knowledge, attitude and practices of the law students on this subject in the international medical literature. 

We therefore conducted this study to compare the knowledge, attitudes, and practices of medical and law students of Erciyes University, Kayseri, Turkey, towards organ donation and transplantation. 

## MATERIAL AND METHODS

This cross-sectional study was conducted in medical and law faculties of Erciyes University, Kayseri, Turkey in 2011. Erciyes University is a state university in the provincial centre of Kayseri, which is located in the central part of Turkey. It was planned to include all 694 students who studied at the first and fourth academic years of medical and law faculties. The study was approved by the ethical committee of Erciyes University Medical Faculty. The administrative permissions were taken from the deaneries of the faculties. 

The obtained data were collected through a questionnaire consisting of 31 questions. The questionnaire was prepared by the researchers in the light of related literature. The questions in the questionnaire were related to socio-demographic characteristics (*eg*, age, sex, school, grade, residence) of the students and their knowledge, attitudes and practices towards organ donation and transplantation. Most of the questions were structured with 2–3 response options. The response options for the structured questions were “yes/no” and “undecided,” if necessary. There was only one open-ended question, about transplantable organs. The answers to this question were evaluated as “know” or “do not know” for some transplantable organs. 

The students in the study group were visited in their classrooms, informed about the study and their verbal consents were taken. All students who were in the classroom accepted to participate in the study. The anonymous questionnaires were distributed to the students. The students filled the questionnaire under the supervision of the researchers. 

The data were analyzed by SPSS^®^ for Windows^®^ ver 15.0. χ^2^ test, *Student’s t* test for independent data, and binary logistic regression analysis were used for statistical analyses. A p value <0.05 was considered statistically significant. 

## RESULTS

Of 694 questionnaires distributed, 498 (71.8%) were evaluated. Of the students, 356 were from medical faculty and 142 from law faculty. Of the study group, 241 (48.4%) were male and 257 (51.6%) were female students. The mean±SD age of participants was 20.4±2.0 years. Of the students, 226 (45.4%) were studying at the first grade of medical faculty, 130 (26.1%) at the fourth grade of medical faculty, 108 (21.7%) at the first grade of law faculty, and 34 (6.8%) at the fourth grade of law faculty. 

It was found that 96.4% of the study group considered that organ transplantation was an important therapeutic method; the frequency was 95.8% for medical students and 97.9% for law students (p=0.489). The level of knowledge the medical and law students about the transplantable organs and tissues is shown in Table 1. The most known transplantable organs were kidney, liver, and heart, respectively. The percentages of the medical students who knew transplantable organs were found significantly higher than that of the law students.

**Table 1:** Comparison of the medical and law students’ knowledge about transplantable organs and tissues

Organ/tissue	Medical faculty (n=356) n (%)	Law faculty (n=142) n (%)	p value
Kidney	318 (89.3)	114 (80.3)	0.007
Hearth	292 (82.0)	93 (65.5)	<0.001
Liver	273 (76.7)	69 (48.6)	<0.001
Cornea	215 (60.4)	52 (36.6)	<0.001
Lung	186 (52.2)	54 (38.0)	0.004
Bone marrow	166 (46.6)	45 (31.7)	0.002
Skin	134 (37.6)	33 (23.2)	0.002
Pancreas	134 (37.6)	32 (22.5)	0.001

There was no significant difference in level of knowledge about transplantable organs between the first grade students of the medical and law faculties. However, the frequency of the students who knew transplantable organs among the fourth grade medical students was significantly higher than that in the fourth grade law students.

The percentage of the students who knew transplantable organs among the fourth grade medical students was significantly higher than that in the first grade. However, no significant difference was found between the first and fourth grade students of the law faculty. 

In the whole study group, 88% of the students knew that transplantation can be performed from the cadaver. This percentage was 85.6% for the medical students and 93.6 % for the law students; 13.5% of the medical students and 29.6% of the law students stated that verbal consent of the individual is sufficient for organ donation. About half of the medical students and 70% of the law students knew where they can apply for organ donation. 

The attitudes of the medical and law students towards organ donation are shown in Table 2. Only 1.4% of the medical students mentioned they had donation card, of whom, 48% thought to donate. On the other hand, none of the law students had donation card; 33.8% of them thought to donate. The difference between the attitudes of the medical and law students towards organ donation was significant. 

**Table 2:** Comparison of the attitudes of the medical and law students towards organ donation

Attitudes towards organ donation	Medical Faculty (n=356) n (%)	Law Faculty (n=142) n (%)	Total (n=498) n (%)
Donated	5 (1.4)	0 (0.0)	5 (1.0)
Want to donate	171 (48.0)	48 (33.8)	219 (44.0)
Do not want to donate	60 (16.9)	37 (26.1)	97 (19.5)
Undecided	120 (33.7)	57 (40.1)	177 (35.5)
Total	356 (100.0)	142 (100.0)	498 (100.0)

χ^2^=12.264, p=0.004

Those students who had donation card and thought to donate were evaluated together and their attitude was interpreted as “positive towards organ donation.” The students who did neither want nor decide to donate organs were also evaluated together and their attitude was interpreted as “negative towards organ donation.” It was found that 45% of the study group had a positive attitude towards organ donation. On the other hand, 81.9% of the students indicated that they would ask for organ from someone, in case they need. The rate was 82.3% for the medical students and 81.0% for the law students (p=0.201). 

The effects of various factors on the attitudes of the students towards organ donation were analyzed through binary logistic regression analysis (Table 3). The percentage of the positive attitude toward organ donation was significantly higher among the medical students than the law students (Table 3); the rate was significantly higher among the fourth grade than the first grade students. Student’s gender, family residence, having a relative waiting for transplantation or transplant recipient did not have any significant effect on his or her attitude. 

**Table 3:** Factors influencing the attitudes of studied students towards organ donation

Independent variables	Group	n	Positive attitude n (%)	OR (95% CI)
Faculty	Law	142	48 (33.8)	1.00
	Medicine	356	176 (49.4)	1.81 (1.18–2.78)
School grade	1^st^ grade	334	128 (38.3)	1.00
	4^th^ grade	164	96 (58.5)	2.15 (1.46–3.19)
Gender	Male	241	99 (41.1)	1.00
	Female	257	125 (48.6)	1.41 (0.97–2.05)
Family residence	Rural	152	59 (38.8)	1.00
	Urban	346	165 (47.7)	1.33 (0.88–2.00)
A relative waiting for transplantation	No	460	199 (43.3)	1.00
	Yes	38	25 (65.8)	2.13 (1.00–4.54)
A relative who is transplant recipient	No	410	174 (42.4)	1.00
	Yes	88	50 (56.8)	1.51 (0.91–2.52)
Total		498	224 (45.0)	

The reasons why students had negative attitude towards organ donation are shown in Table 4; 21.3% of the medical students, and 12.7% of the law students stated that their knowledge about medical aspects of organ donation and transplantation was sufficient. However, the percentage of students who knew legal aspects of organ donation and transplantation was 14.3%, and 54.9% respectively. The difference between medical and law students in terms of their self-evaluations was significant. Of studied students, 85.1% of medical students and 84.5% of law students stated they wanted more information about organ donation and transplantation. 

**Table 4:** Comparison of the study groups according the reasons of the students who did not want or were uncertain to donate organs.

Reasons for not donating organ	Medical faculty (n=356) n (%)	Law faculty (n=142) n (%)
Organ donation is not appropriate religiously	29 (16.3)	12 (12.8)
Afraid of taking organs before the death	42 (23.3)	29 (30.9)
Does not want disruption of body integrity after the death	37 (20.6)	19 (20.2)
Fear that their organs will be given to those who do not want	12 (6.7)	4 (4.3)
Fear of commercial exploitation of organs	26 (14.4)	15 (16.0)
Others	34 (18.9)	15 (16.0)
Total	180 (100.0)	94 (100.0)

χ^2^=2.818, p=0.728

## DISCUSSION

Necessary organs for transplantation can be obtained from cadaver or live donor. In most western countries, organ transplantations are mainly performed from cadavers. However, in Turkey, the situation is different and transplantations are mostly performed from live donors [17, 18].

The most well-known transplantable organs are kidney, heart, and liver. The order of the most well-known transplantable organs by the students in this study was similar to other studies earlier performed in Turkey and in other countries [3, 10, 13, 14, 19, 20]. This situation might depend on the fact that kidney transplantation is the most commonly performed organ transplantation in Turkey, in particular, and in the world, at large. 

We found that the percentage of students who knew each of the transplantable organs was significantly higher among medical students compared to the law students. When the first and fourth grade students were evaluated separately in terms of their knowledge about transplantable organs, we found there was no difference between the first year medical and law students, whereas the knowledge of the fourth year medical students was significantly higher than that of the fourth year law students. These differences may be due to the presence of topics in the third and fourth years of medical curriculum and the higher likelihood that a fourth year medical student meets a patient with organ failure or transplant. In a study conducted on medical students in Brazil, it was found that knowledge of the students about transplantable organs was higher during the later years of the study [10].

Of the medical students studied, 1.4% stated they had donation card, of whom, 49.4% were willing to donate. On the other hand, among the law students, there was no one who had donation card and the percentage of those who were willing to donation was 33.8%. The percentage of the students who had donation card and those who were willing to donate varies from study to study. While the percentage of the students who had donor card was quite high in some countries, it was very low in Turkey. The percentage of medical students with donor card was 80% in the USA, 69.2% in Brazil, 43% in the UK, 31.9% in Germany, 22% in Iran, 8.7% in Greece, and 8% in South Africa [8, 10, 11, 14, 16, 20, 21]. According to previous studies conducted in Turkey, the proportion of people who had donation card varied between 1.8% and 13.4% [22–24]. 

The percentages of having donor card were generally higher in among medical students than in other students. In a study conducted in Germany, 31.0% of medical students owed donor card; the rate was 11.3% among other students [20]. In a study conducted in Greece, the percentages were 8.7%, and for medical students, 3.6% for nursing students, and 3.1% for medical laboratory students [14]. In contrast, in a study in England, 74% of nursing students and 43% of medical students had donation card [21].

The proportion of people who were willing organ donation mentioned differently between 30% and 91%, in various studies. [3, 19, 22, 23, 25]. The percentage of potential organ donors was lower among law students compared to medical students. This difference may be due to the fact that medical students encounter more frequently with patients in need of organ transplantation than law students. On the other hand, there are some issues about organ donation and transplantation in the third and fourth years of the curriculum of medical faculty. 

Organ donation can be done in provincial health directorates, security directorates, hospitals, organ transplantation centers, foundations related to organ donation, *etc*. According to a study held by Naçar, *et al* [19], 48.5% of medical students knew where to apply for organ donation. In our study, 50.8% of medical students and 69.0% of law students knew where to apply. These data show that law students are better informed than medical students on legal aspects of donation.

Almost half of the medical students and one-third of law students stated that they considered organ donation. On the other hand, 82.3% of medical students and 81.0% of law students expressed their willingness to receive organ or tissue from another person, if they need. In a study held by Baykan, *et al* [26], on the first year of medical students, similar results were found. These data show that organ transplantation is acknowledged by the majority of the people as a therapeutic method, but participation to organ donation has not yet reached the desired level.

In our study, when the reasons of the students who did not want to donate or who were uncertain about the matter were asked, it was found that 27.7% of the students were afraid of removing their organs before death, 21.9% did not want their physical integrity to be disrupted after the death, 16.0% found organ donation religiously inappropriate, and 16.0% were anxious about being commercially abused. Almost 18% of medical students and 13.3% of law students were those who supposed organ donation was not comply with their religion. According to a study carried out by Özmen, *et al* [3], on health college students, 38.2% of the students who did not consider donation, stated that they did not want their physical integrity to be disrupted, 41.2% was conscientiously uncomfortable, 17.6% thought that donated organs would be abused commercially, and 29.4% found organ donation religiously improper.

The percentage of the students who found their knowledge on medical aspects of organ transplantation sufficient was higher among medical students; however, more law students thought their knowledge on legal aspects of the matter is sufficient. Majority of both groups found their knowledge on both aspects was insufficient. Approximately 85% of the students in both groups indicated that they wanted to learn more about organ donation and organ/tissue transplantation. 

This investigation is one of the first studies to compare the knowledge, attitudes and practices of medical and law students toward organ donation. The study has some limitations. The study was performed in a single university and thus, the results may not be generalized to all medical and law students in Turkey.

In conclusion, we found that medical and law students have insufficient information about transplantation and organ donation. The needs for organ donation have been increasing in all communities. Education programs, which provide information about transplantation and organ donation for all sectors of the community, should be prepared; these programs should be applied regularly in all educational institutions from elementary schools. Programs about organ donation should be prepared and shown in the media, especially on television and on the Internet.

To raise awareness among students, education related to organ donation should be enhanced and sustained. Education programs can be carried out to motivate those who are uncertain about donation. For example, a documentary about the patients who are waiting for organs can be shown to all students.

## FOUNDING:

The authors declared that no financial support was taken for this study.
